# Effects of E-Cigarette Aerosol Extracts on Human Neutrophil Viability and Inflammatory Responses to *Streptococcus mutans*

**DOI:** 10.3390/dj14100645

**Published:** 2026-10-07

**Authors:** Abedelmalek Kalefh Tabnjh, Peter Lingström, Guglielmo Campus, Karin Christenson, Anna-Karin Östberg, Anders Blom, Anne Farbrot, Sara Alizadehgharib

**Affiliations:** 1 Department of Cariology, Institute of Odontology, Sahlgrenska Academy, University of Gothenburg, 405 30 Gothenburg, Sweden; lingstrom@odontologi.gu.se (P.L.); guglielmo.campus@gu.se (G.C.); 2 Department of Applied Dental Sciences, Faculty of Applied Medical Sciences, Jordan University of Science and Technology, Irbid 22110, Jordan; 3 Department of Oral Microbiology and Immunology, Institute of Odontology, Sahlgrenska Academy, University of Gothenburg, 405 30 Gothenburg, Sweden; karin.christenson@gu.se (K.C.); anna-karin.ostberg@odontologi.gu.se (A.-K.Ö.); sara.alizadehgharib@gu.se (S.A.); 4 Department of Occupational and Environmental Medicine, Sahlgrenska University Hospital, 405 30 Gothenburg, Sweden; anders.p.blom@vgregion.se (A.B.); anne.farbrot@vgregion.se (A.F.)

**Keywords:** dental caries, e-cigarettes, electronic smoking, flavors, IL-1β, IL-8, neutrophils, proinflammatory markers, vaping

## Abstract

**Background/Objective:** The increasing use of electronic cigarettes (e-cigarettes) has raised concerns about their possible effects on oral immune responses. Neutrophils are essential for host defense against cariogenic bacteria such as *Streptococcus mutans* (*S. mutans*), and impaired neutrophil function may facilitate the progression of oral diseases. This study aims to investigate whether exposure to vaping aerosols alters neutrophil viability, oxidative responses, and proinflammatory cytokine production in response to the cariogenic bacterium *S. mutans*. **Methods:** Human neutrophils isolated from healthy donors were exposed to e-cigarette aerosol extracts produced from flavored and unflavored e-liquids, with and without nicotine. Cytotoxicity was determined by measuring lactate dehydrogenase (LDH) release. Intracellular reactive oxygen species (ROS) generation following *S. mutans* exposure was used as an indirect measure of neutrophil phagocytic activity. Pro-inflammatory cytokines (interleukin-1 beta (IL-1β) and interleukin-8 (IL-8)) were measured using an enzyme-linked immunosorbent assay (ELISA). **Results:** Under the tested conditions, e-cigarette aerosol extracts did not cause significant cytotoxicity. However, in neutrophils stimulated with *S. mutans*, certain flavorings (particularly tobacco and dessert-related flavors) significantly reduced ROS generation, suggesting impaired antimicrobial responses. In contrast, fruit and mint flavors had little impact. Minor flavor-dependent differences were observed for IL-1β and IL-8. The presence of nicotine did not significantly affect the levels of these cytokines. **Conclusions:** Aerosol extracts from e-cigarettes altered neutrophil oxidative responses in a flavor-dependent manner but did not cause significant cytotoxicity. These findings suggest mild immunological modulation rather than a direct inflammation effect and indicate that e-cigarette aerosols may alter antimicrobial responses despite having minimal effects on IL-1β and IL-8.

## 1. Introduction

According to the World Health Organization (WHO), the use of electronic cigarettes (e-cigarettes), commonly referred to as vaping, has increased substantially, particularly among adolescents and young adults. It is now considered an emerging public health concern in many countries [1]. It is estimated that more than 100 million people worldwide use e-cigarettes, with the highest prevalence reported in Europe. Adolescents are three times more likely than adults to use e-cigarettes [2]. Although e-cigarettes are commonly used as an alternative to conventional cigarettes, their use is not without health risks [3].

The oral cavity is the primary site of exposure to e-cigarette aerosols, which contain a complex mixture of substances including propylene glycol (PG), vegetable glycerin (VG), nicotine, and flavoring agents. Upon heating, these components can generate reactive by-products such as aldehydes and metals, which may interact with oral tissues and the dental biofilm [4,5,6,7,8,9]. In addition to their direct effects on oral structures, these compounds may alter local immune responses and contribute to inflammatory processes within the oral environment.

Emerging evidence indicates that e-cigarette exposure can induce oxidative stress and inflammatory responses, as well as alter immune cell function [10]. Flavoring composition and nicotine concentration may modulate the biological effects of e-liquids, with some flavoring chemicals demonstrating cytotoxic effects in vitro [11]. However, the extent to which nicotine and flavoring agents contribute to these biological effects is still unclear.

Neutrophils are key components of the innate immune system and play a central role in host defense against microbial pathogens. Upon activation, they can generate reactive oxygen species (ROS) and release cytotoxic mediators that contribute to pathogen clearance. Dysregulation of these processes may lead to tissue damage and chronic inflammation [12]. In the oral cavity, neutrophils are essential for controlling bacterial biofilms, including cariogenic species such as *Streptococcus mutans* (*S. mutans*), which significantly contribute to the development of dental caries. *S. mutans* interacts with host immune cells within dental biofilms and the gingival crevice, where neutrophils constitute an important component of the first-line defense. Consequently, alterations in neutrophil function may impair bacterial clearance and dysregulate inflammatory responses, potentially modifying host–microbe interactions relevant to oral diseases, including dental caries.

Inflammatory cytokines such as interleukin-1 beta (IL-1β) and interleukin-8 (IL-8) are important mediators of oral immune responses. IL-1β is associated with tissue destruction and bone resorption, while IL-8 regulates neutrophil recruitment and activation in gingival tissues [13,14]. IL-1β is a critical mediator of acute inflammation, and elevated levels have been reported in the saliva and dental tissues of persons with caries, indicating a positive correlation with disease occurrence and progression [15]. Similarly, exposure to bacterial antigens can stimulate immune cells to release IL-8, a chemokine that helps attract neutrophils [16,17]. Elevated levels of IL-8 have also been documented in individuals with dental caries, supporting its role as an indicator of active inflammation [18]. Exploring the release of IL-1β and IL-8 following exposure to e-cigarette extracts provides important insights into the pro-inflammatory potential of vaping within the oral environment [19]. Changes in cytokine production may reflect alterations in host–microbe interactions and be associated with the development of oral diseases.

Despite the growing body of research on e-cigarettes, significant knowledge gaps remain regarding their impact on immune cell function within the oral environment. In particular, limited information is available on whether e-cigarette aerosols with different flavor profiles and nicotine content differentially modulate human neutrophil responses to cariogenic bacteria. Addressing this gap is important because flavoring constituents and nicotine may independently influence innate immune functions relevant to oral host–microbe interactions. Therefore, the aim of this study was to investigate and compare the effects of flavored and unflavored e-cigarette aerosol extracts, with and without nicotine, on neutrophil viability, oxidative responses, and pro-inflammatory cytokine production following stimulation with the cariogenic bacterium *S. mutans*.

## 2. Materials and Methods

Aerosol extracts were generated from e-liquids with or without nicotine and with different flavorings. The cytotoxic effects of these extracts on neutrophils were assessed by measuring lactate dehydrogenase (LDH) release. Intracellular reactive oxygen species (ROS) production was analyzed as an indicator of neutrophil activation and phagocytic function. In addition, the secretion of IL-1β and IL-8 was quantified using an enzyme-linked immunosorbent assay (ELISA) following exposure to e-cigarette aerosol extracts alone and in combination with *S. mutans*.

### 2.1. E-Cigarette Aerosol Extract Preparation

Commercially available e-liquids were purchased from commercial eiers in Sweden in February 2025. All e-liquids were prepared with a 50:50 ratio of propylene glycol (PG) to vegetable glycerin (VG). In this study, e-liquids with different flavors were used, either nicotine-free or containing 14 mg/mL of nicotine. The e-liquids were from the fourth generation of e-cigarettes, which have cooling agents and use nicotine salts rather than free-base nicotine. A total of 14 samples were selected to represent unflavored and commonly used flavored liquids (Table 1).

E-cigarette extracts were prepared in the AstraZeneca smoking laboratory (Mölndal, Sweden). Briefly, 20 mL of phosphate-buffered saline (PBS) was added to the collection flask, and the airflow was adjusted to 0.95 L/min. The timer was set to the required extraction time, and aerosols generated from the e-liquids were bubbled through the PBS solution. Continuous bubbling was visually confirmed throughout aerosol generation. E-liquids were heated into aerosols using an electronic nicotine delivery system (ENDS). The puff duration was set to 3 s following a standardized protocol of 3 puffs per milliliter of PBS, resulting in a total of 60 puffs for 20 mL of PBS. The aerosol generation parameters were selected based on puffing parameters reported in previous e-cigarette studies [20]. A puff duration of 3 s was used to approximate commonly reported e-cigarette puffing behavior [21]. The standardized exposure of three puffs per milliliter of PBS, corresponding to 60 puffs collected in 20 mL of PBS, was used to generate a reproducible aerosol extract of sufficient concentration for the subsequent in vitro experiments. However, this experimental exposure should not be interpreted as a direct quantitative equivalent of a specific number of puffs or duration of vaping in humans. After the aerosol was collected, the extracts were stored at −80 °C until further analysis.

### 2.2. Isolation of Human Neutrophils

Neutrophils were obtained from de-identified buffy coats of healthy blood donors provided by the Sahlgrenska University Hospital blood bank. Donors fulfilled the eligibility criteria for routine blood donation, and no additional study-specific inclusion or exclusion criteria were applied. Donor-identifying or detailed clinical information was not available to the researchers. Cells from each donor were processed individually. Neutrophils were isolated using dextran sedimentation, followed by density gradient centrifugation and hypotonic lysis.

Briefly, buffy coats were diluted in NaCl containing dextran and incubated for 30–40 min at room temperature to allow erythrocyte sedimentation. The leukocyte-rich supernatant was collected and carefully layered over Ficoll-Paque (Cytiva, Uppsala, Sweden), followed by centrifugation at 930× *g* for 15 min at 4 °C. After removing the peripheral blood mononuclear cell (PBMC) layer and Ficoll phase, the granulocyte-containing pellet was retained.

From this step onward, all procedures were performed on ice. Residual erythrocytes were removed by hypotonic lysis using distilled water, after which isotonicity was restored with PBS/NaCl. Cells were washed 2–3 times in Krebs–Ringer glucose buffer (KRG) and centrifuged between washes. Finally, cells were resuspended in KRG supplemented with calcium (1 mM). Neutrophils were counted and adjusted to a final concentration of 1 × 10^7^ cells/mL and maintained on ice until use.

### 2.3. E-Cigarette Aerosol Extracts Cytotoxicity

Cytotoxicity was assessed by measuring lactate dehydrogenase (LDH) release from neutrophils using the Cytotoxicity Detection kit LDH (Roche Diagnostics GmbH, Mannheim, Germany). Neutrophils were diluted to a final concentration of 4 × 10^6^ cells/mL, and 50 µL of the cell suspension (2 × 10^5^ cells) was distributed into each well of a 96-well plate. E-cigarette aerosol extracts were tested at final concentrations of 3.125%, 6.25%, 12.5%, 25%, 50%, and 75%. The total reaction volume was 250 µL per well and consisted of extract, neutrophils, and RPMI medium (Gibco, Paisley, UK) supplemented with 1% penicillin-streptomycin (PEST) and 10% fetal calf serum (FCS).

Control wells contained medium alone, freshly separated cells without addition of aerosol extracts, frozen cell supernatants (supernatants collected before freezing) without addition of aerosol extracts, or neutrophils treated with Triton X-100 to achieve maximal LDH release. After incubation for 24 h, 75 µL of supernatant from each well was transferred and mixed with 75 µL of LDH detection reagent. The reaction was allowed to proceed for 10 min at room temperature in the dark, and then absorbance was measured at 490 nm using a CLARIOstar Plus microplate reader (BMG LABTECH, Ortenberg, Germany).

All conditions were analyzed in duplicate. Eight independent experiments were performed using neutrophils isolated from eight different buffy coats.

### 2.4. Flow Cytometric Analysis to Evaluate Cell Death

To confirm the LDH results, a flow cytometric analysis of cell death was performed. Isolated human neutrophils (50 µL, 5 × 10^6^ cells/mL) (n = 3) were suspended in RPMI medium (Gibco, Paisley, UK) complemented with 10% FCS and 1% PEST and incubated for 30 min at 37 °C in 5% CO_2_. The cells were then exposed to e-cigarette aerosol extracts at two concentrations: one at a high concentration (75%) and the other at the lowest concentration (3.125%). LPS (10 μg/mL; Sigma-Aldrich, St. Louis, MO, USA) was used as a negative control (anti-apoptotic), and pro-apoptotic anti-CD95 (Becton Dickinson, Mountain View, CA, USA) (20 μg/mL) was used as a positive control. Duplicate samples were used as a control containing only cells and RPMI.

After 20 h of incubation, all cultured samples were washed in 2 mL of Annexin buffer [1 mM of Hepes, 14 mM of NaCl, and 0.25 mM of CaCl_2_, (pH 7.4)]. The pellets were resuspended in 102 μL of Annexin buffer, supplemented with 2 μL of Annexin V-FLUOS and 5 μL of 7-amino-actinomycin D (7-AAD) (Becton Dickinson, Mountain View, CA, USA), and incubated for 10 min in the dark. Subsequently, an additional 300 μL of Annexin buffer was added, and samples were subjected to FACS analysis using an Accuri C6 (Becton Dickinson, Mountain View, CA, USA). At least 10,000 events were acquired, and neutrophils were gated based on side- and forward-scatter. Apoptosis was assessed based on Annexin V-FLUOS fluorescence, and necrosis was assessed based on membrane permeability to 7-AAD.

### 2.5. Measurement of Intracellular Reactive Oxygen Species (ROS)

Intracellular ROS production was measured as an indicator of neutrophil oxidative responses, providing an indirect measurement of neutrophil phagocytic activity following stimulation with *S. mutans*. However, ROS generation does not directly measure bacterial phagocytosis or bactericidal capacity and should therefore be interpreted as an indicator of neutrophil oxidative responses rather than a direct measure of bacterial uptake or killing.

The intracellular generation of ROS in neutrophils was measured using luminol-enhanced chemiluminescence. Neutrophils (5 × 10^5^) were added to a 96-well plate. Cells were exposed to e-cigarette aerosol extracts (25%), *S. mutans* (100 bacteria per cell, opsonized with 10% human serum), or phorbol 12-myristate 13-acetate (PMA, 50 nM) as a positive control. The 25% aerosol extract concentration was selected based on the LDH experiments as a non-cytotoxic experimental exposure suitable for assessing functional neutrophil responses. In this in vitro model, 25% refers to the proportion of the standardized PBS aerosol extract in the final experimental volume and does not represent a defined aerosol concentration or dose in the human oral cavity. Appropriate buffer controls were included. A luminol-based master mix containing luminol, superoxide dismutase (SOD, 500 U/mL), and catalase (2000 U/mL) (Worthington Biochemical Corporation, Lakewood, NJ, USA) was added to each well to a final volume of 200 µL. The plate was incubated at 37 °C for 5 min prior to measurement. Baseline chemiluminescence was recorded before the addition of stimuli, after which ROS production was monitored for approximately 90 min using a plate reader. Statistical comparisons were performed at 45 min, when ROS production had reached peak levels.

### 2.6. Pro-Inflammatory Cytokine Analysis

Following isolation from human buffy coats, neutrophils were seeded at a concentration of 5 × 10^5^ cells per well in 96-well plates containing RPMI medium (Gibco, Paisley, UK) supplemented with 10% FCS. Cells were exposed to e-cigarette aerosol extracts in the presence of *S. mutans*. Three control conditions were included: neutrophils alone or stimulated with *S. mutans* (control) or lipopolysaccharide (LPS) as a positive control.

Following incubation for 20–24 h, plates were frozen at −80 °C. After thawing, samples were centrifuged, and the cell-free supernatants were collected and stored at −80 °C until analysis of pro-inflammatory cytokines. IL-8 and IL-1β levels were measured using an ELISA according to the manufacturer’s instructions (Invitrogen, by Thermo Fisher Scientific, Vienna, Austria).

### 2.7. Chemical Characterization of E-Cigarette Aerosol Extracts

A 500 µL aliquot of each e-cigarette aerosol extract was transferred to a clean glass vial. For extraction, an equal volume of dichloromethane was added, and the vial was vigorously shaken for 30 min. Following a settling period of one hour, phase separation was achieved, and an aliquot of the dichloromethane phase was transferred to a separate vial for analysis.

Samples were analyzed using a gas chromatography–mass spectrometry (GC-MS) system (7890A/5975C; Agilent Technologies, Inc., Santa Clara, CA, USA) equipped with a 30 m × 0.25 mm × 0.25 µm capillary column (Macherey-Nagel MN-5 Accent). The oven temperature program consisted of a linear ramp of 10 °C/min from an initial temperature of 35 °C to a final temperature of 330 °C. Helium was used as the carrier gas at a flow rate of 1.2 mL/min. A 2 µL aliquot was injected in pulsed splitless mode. Mass spectral data were acquired in scan mode over the range of 38–350 m/z. Ionization was performed using standard electron ionization (EI) conditions to obtain searchable mass spectra.

Data were processed using Automated Mass Spectral Deconvolution and Identification System (AMDIS) deconvolution software (version 2.70) and searched against the National Institute of Standards and Technology (NIST) 14 mass spectral library. For each detected component, the five highest-ranking library matches were evaluated, and the most appropriate match was selected for reporting.

The identified compounds were subsequently compared across the different flavor categories, and the results were visualized using a heatmap. The heatmap was based on semi-quantitative abundance categories assigned during the GC-MS data evaluation. For visualization purposes, these abundance categories were converted to a normalized scale ranging from 0 to 100. The resulting values represent relative abundance indices only and should not be interpreted as quantitative measurements of compound concentrations.

### 2.8. Statistical Analysis

Data were analyzed using GraphPad Prism version 9.5.1 (GraphPad Software, LLC, San Diego, CA, USA). As measurements obtained from the same buffy coat donor were considered paired, comparisons involving multiple related conditions were performed using the Friedman test, followed by Dunn’s multiple-comparison test for post hoc pairwise comparisons. Dunn’s test included adjustment for multiple comparisons. Depending on the analysis, comparisons were performed between individual aerosol extracts and the corresponding control, between flavor groups, or between nicotine-containing and nicotine-free extracts. Data are presented as the mean ± SEM unless otherwise stated. Statistical significance was defined as *p* < 0.05 and is consistently reported using the following ranges: * *p* < 0.05, ** *p* < 0.01, *** *p* < 0.001, and **** *p* < 0.0001.

## 3. Results

### 3.1. E-Liquid Aerosol Extract Cytotoxicity

**Figure 1:**
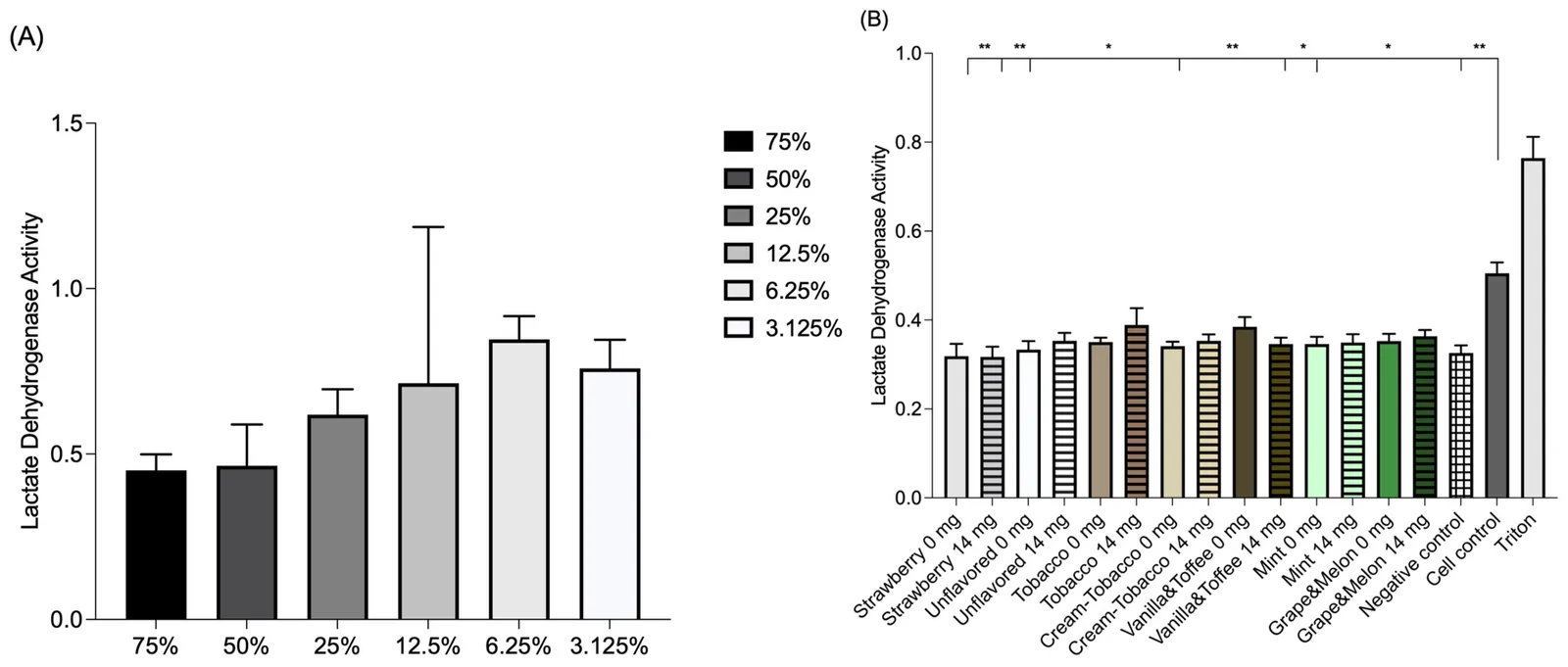
LDH release from human neutrophils following exposure to e-cigarette aerosol extracts. (**A**) Concentration-dependent LDH release following exposure to pooled e-cigarette aerosol extracts at different concentrations. (**B**) LDH release following exposure to individual flavored e-cigarette aerosol extracts (25%) with and without nicotine after 24 h incubation. Negative frozen control represents neutrophil supernatants frozen immediately after cell isolation to reflect minimal spontaneous LDH release. Cell control represents neutrophils cultured in medium alone for 24 h. Triton means Triton X-100-treated cells used as a positive control for maximal LDH release. Data are presented as the mean ± SEM from eight independent experiments (n = 8). Statistical significance is indicated as * *p* < 0.05 and ** *p* < 0.01.

Cytotoxicity of the e-cigarette aerosol extracts was assessed by measuring LDH release from neutrophils exposed to extracts generated from flavored and unflavored e-liquids, with or without nicotine. Interestingly, LDH release decreased with increasing concentrations of the e-cigarette aerosol extracts, showing an inverse concentration-dependent pattern (Figure 1A). Based on the LDH results, a 25% extract concentration was selected for subsequent functional experiments because it represented a non-cytotoxic experimental exposure while providing sufficient aerosol extract for assessing potential effects on neutrophil function (Figure 1A). As shown in Figure 1B, most extracts induced a relatively low LDH release compared to the control sample (neutrophils cultured in medium without extract). LDH levels were comparable to those observed in the frozen cell supernatant control (negative control) and the medium-only control, indicating minimal cytotoxicity under the tested conditions.

**Figure 2:**
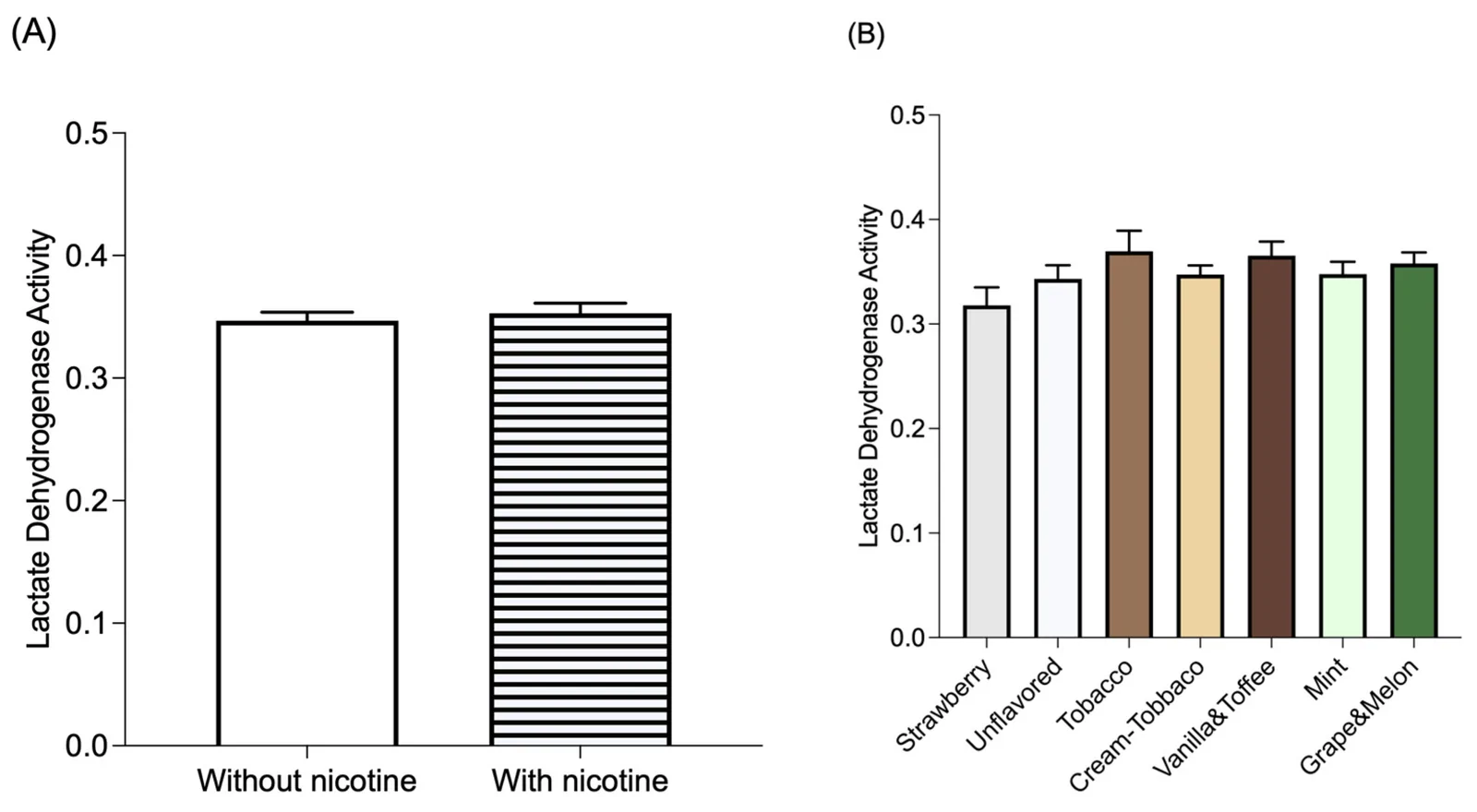
Lactate dehydrogenase (LDH) release from neutrophils following exposure to different flavored e-cigarette aerosol extracts (25%) (**A**) and in pooled samples with or without nicotine (**B**). LDH levels are expressed as mean ± SEM.

Comparing the effects of different flavors revealed no significant differences in LDH release between flavored and unflavored e-cigarette aerosol extracts, with similar LDH levels across the tested conditions. No significant differences were observed between nicotine-containing and nicotine-free extracts, indicating that nicotine did not influence neutrophil membrane integrity under the tested conditions (Figure 2).

### 3.2. Flow Cytometric Analysis of Neutrophil Viability

**Figure 3:**
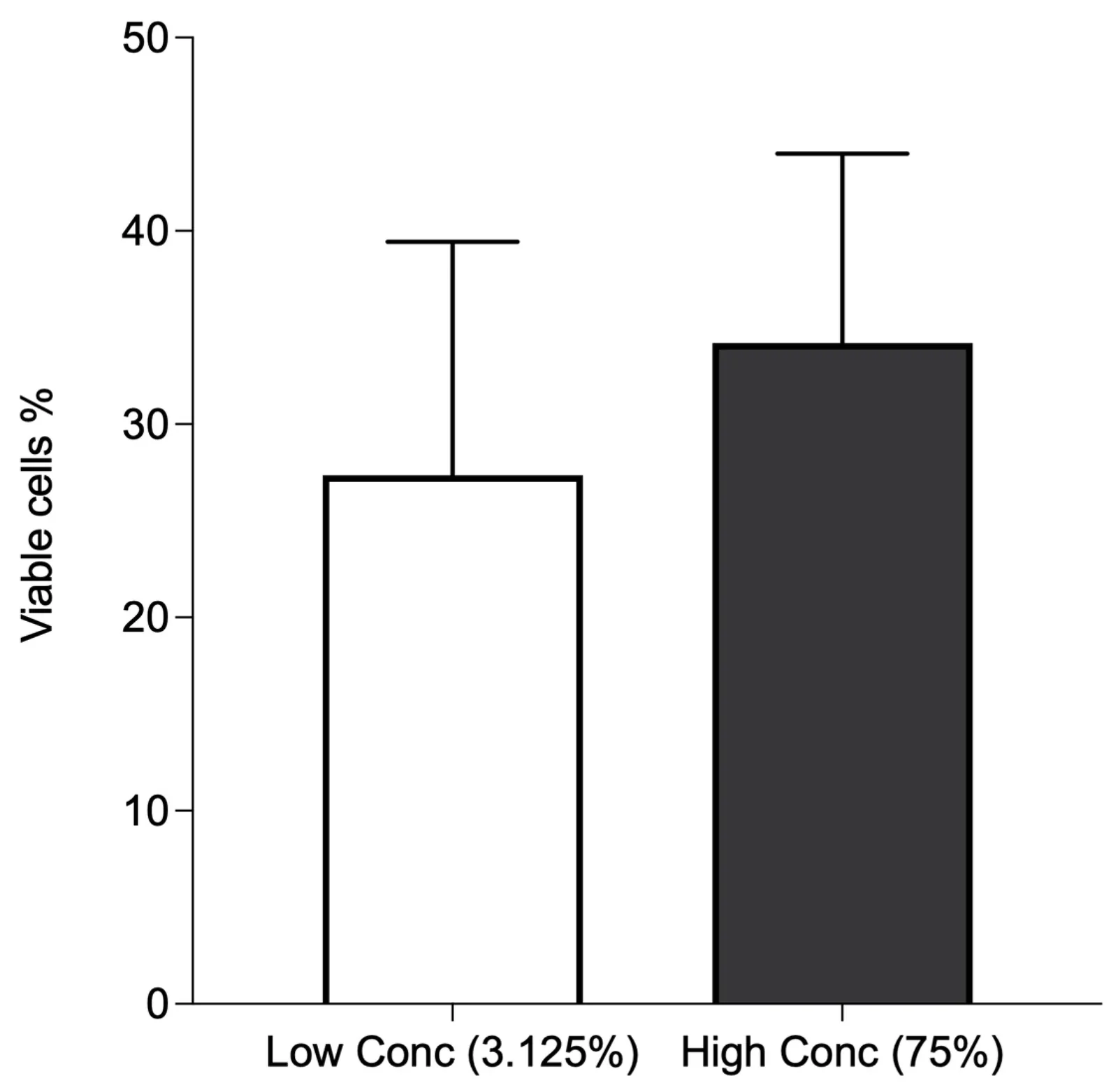
Flow cytometric analysis of neutrophil viability following exposure to e-cigarette aerosol extracts. Human neutrophils were exposed to pooled flavored e-cigarette aerosol extracts (including both products with and without nicotine) at low (3.125%) and high (75%) concentrations for 20 h. Cell viability was assessed using Annexin V and 7-AAD staining and analyzed using flow cytometry. The figure shows the percentage of viable neutrophils (Annexin V−/7-AAD−). Data are presented as the mean ± SEM from three independent experiments (n = 3).

To confirm the LDH findings, neutrophil viability was further assessed by flow cytometry following exposure to e-cigarette aerosol extracts at the lowest (3.125%) and highest (75%) extract concentrations. Cell populations were classified as viable, apoptotic, or necrotic based on Annexin V and 7-AAD staining. The proportion of viable neutrophils appeared slightly higher following exposure to 75% extract compared with 3.125% extract (Figure 3). However, given the small sample size (n = 3), these findings should be interpreted with caution, and no formal statistical conclusions were drawn.

### 3.3. Intracellular Reactive Oxygen Species (ROS)

**Figure 4:**
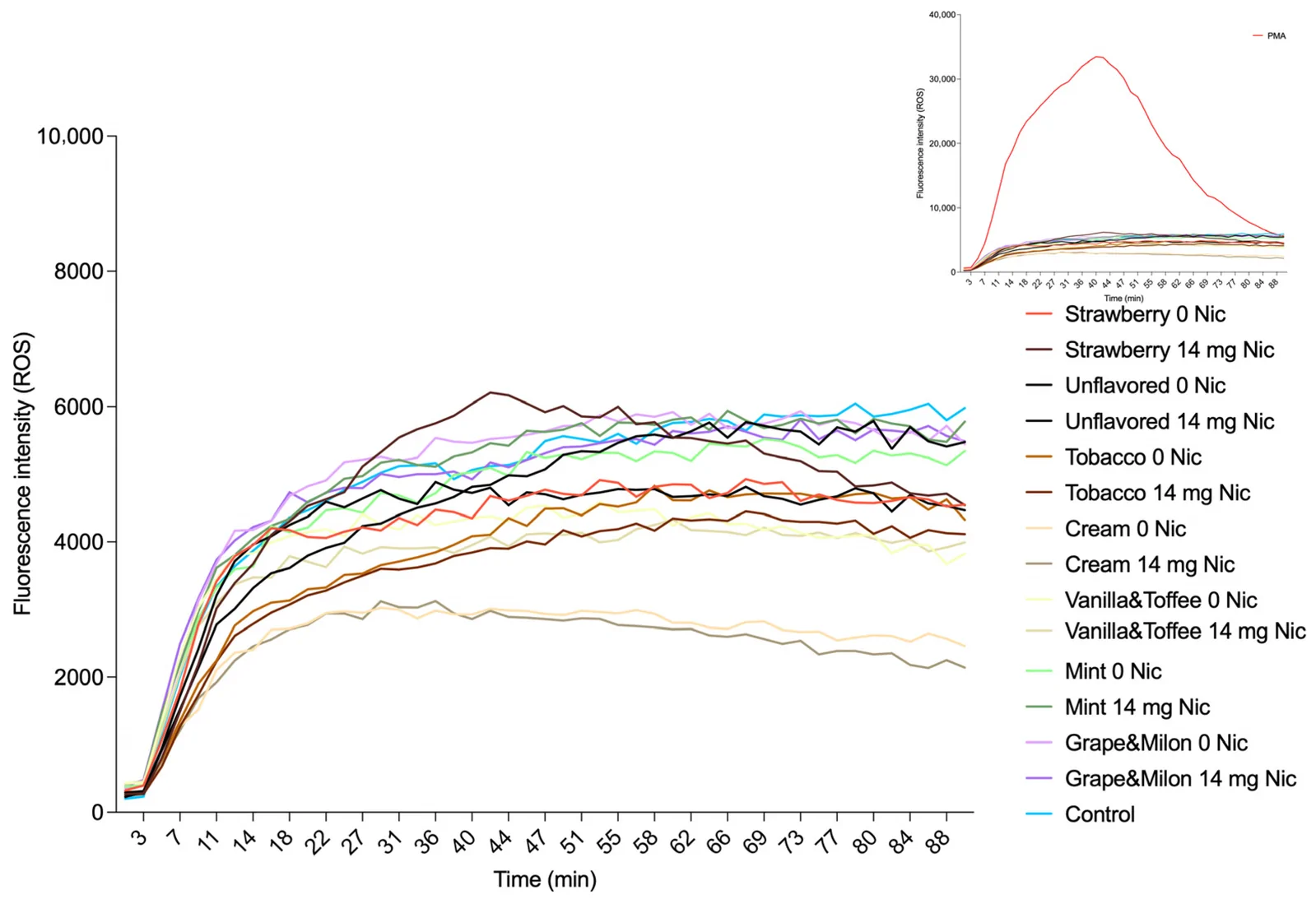
Time course of intracellular reactive oxygen species (ROS) production in human neutrophils following exposure to e-cigarette aerosol extracts and stimulation with *Streptococcus mutans* (*S. mutans*). Neutrophils were exposed to 25% aerosol extracts and stimulated with opsonized *S. mutans* or *S. mutans* alone (control). Intracellular ROS production was monitored over time by luminol-enhanced chemiluminescence. PMA (50 nM) was used as a positive control for ROS generation and is shown in the inset. The data represents mean values from eight independent experiments (n = 8).

**Figure 5:**
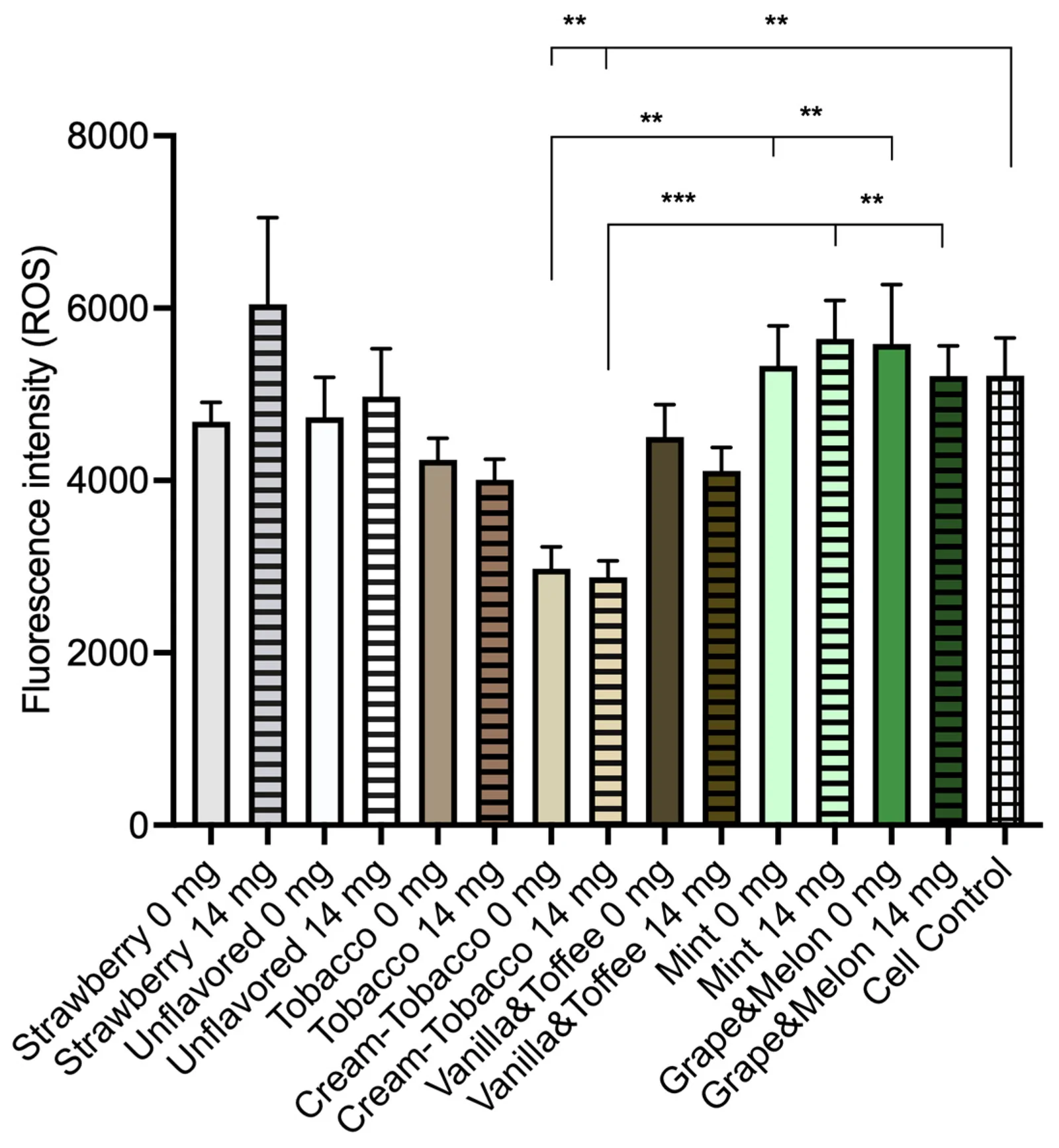
Effects of different e-liquid aerosol extracts on intracellular reactive oxygen species (ROS). Human neutrophils were exposed to *Streptococcus mutans* (*S. mutans*) and to different aerosol extracts. Cell control included cells exposed to *S. mutans* without aerosol extract. The data are shown as the mean peak values ± SEM at 45 min, and the level of significance is indicated as ** < 0.01 and *** < 0.001 (n = 8).

The intracellular production of ROS in neutrophils stimulated with *S*. *mutans* was measured kinetically following exposure to different e-cigarette aerosol extracts (conc. of 25%) (Figure 4). As shown in Figure 5, several extracts affected the intracellular ROS production compared with the control (neutrophils stimulated with *S. mutans* in the absence of extracts). Among the tested samples, the cream-tobacco flavor induced a statistically significant decrease in ROS levels (*p* < 0.01).

**Figure 6:**
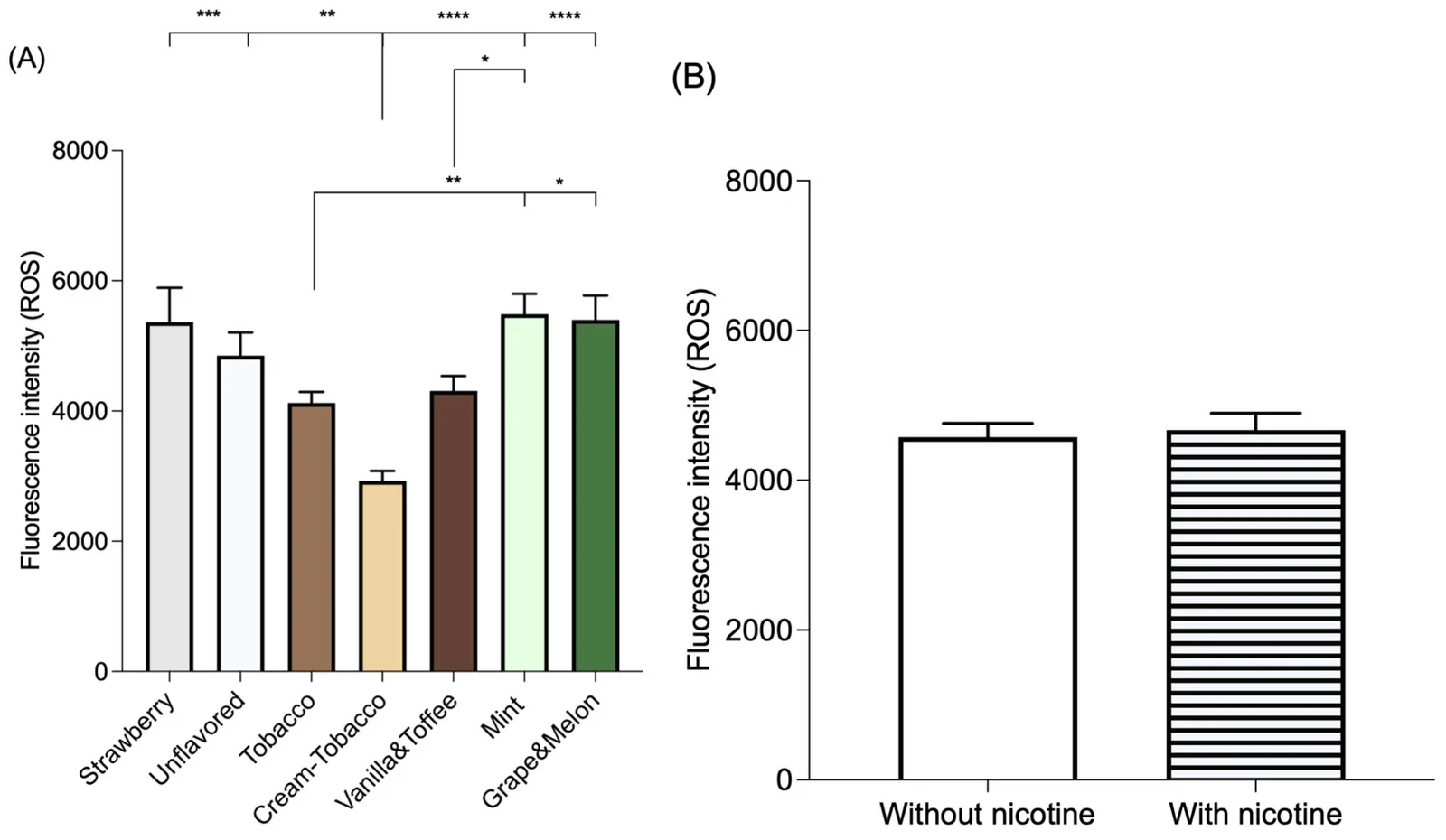
Comparison of different flavors of e-cigarette aerosol extracts (**A**) and in pooled samples with and without nicotine (**B**) on intracellular reactive oxygen species (ROS) production in human neutrophils at 45 min following phagocytosis of *Streptococcus mutans*. The data are shown as the mean peak values ± SEM, and the level of significance is indicated as * 0.05, ** 0.01, *** 0.001, and **** 0.0001 (n = 8).

When the extracts were classified based on flavor, significant differences in ROS production were observed between several flavor categories. Cream-tobacco induced significantly lower ROS production compared with strawberry, unflavored, mint, and grape and melon. Tobacco also induced significantly lower ROS production compared with mint and grape and melon. In addition, vanilla and toffee induced significantly lower ROS production compared with mint. No significant differences were observed among the remaining flavor comparisons (Figure 6A). Although reduced ROS production by neutrophils was observed with both nicotine-containing and nicotine-free extracts, no statistically significant effect of nicotine was observed (Figure 6B). These findings suggest that e-cigarette aerosol extracts induce flavor-dependent effects, especially tobacco and dessert flavors, on intracellular ROS production in neutrophils exposed to *S. mutans*, regardless of nicotine content.

### 3.4. Pro-Inflammatory Cytokine Analysis

**Figure 7:**
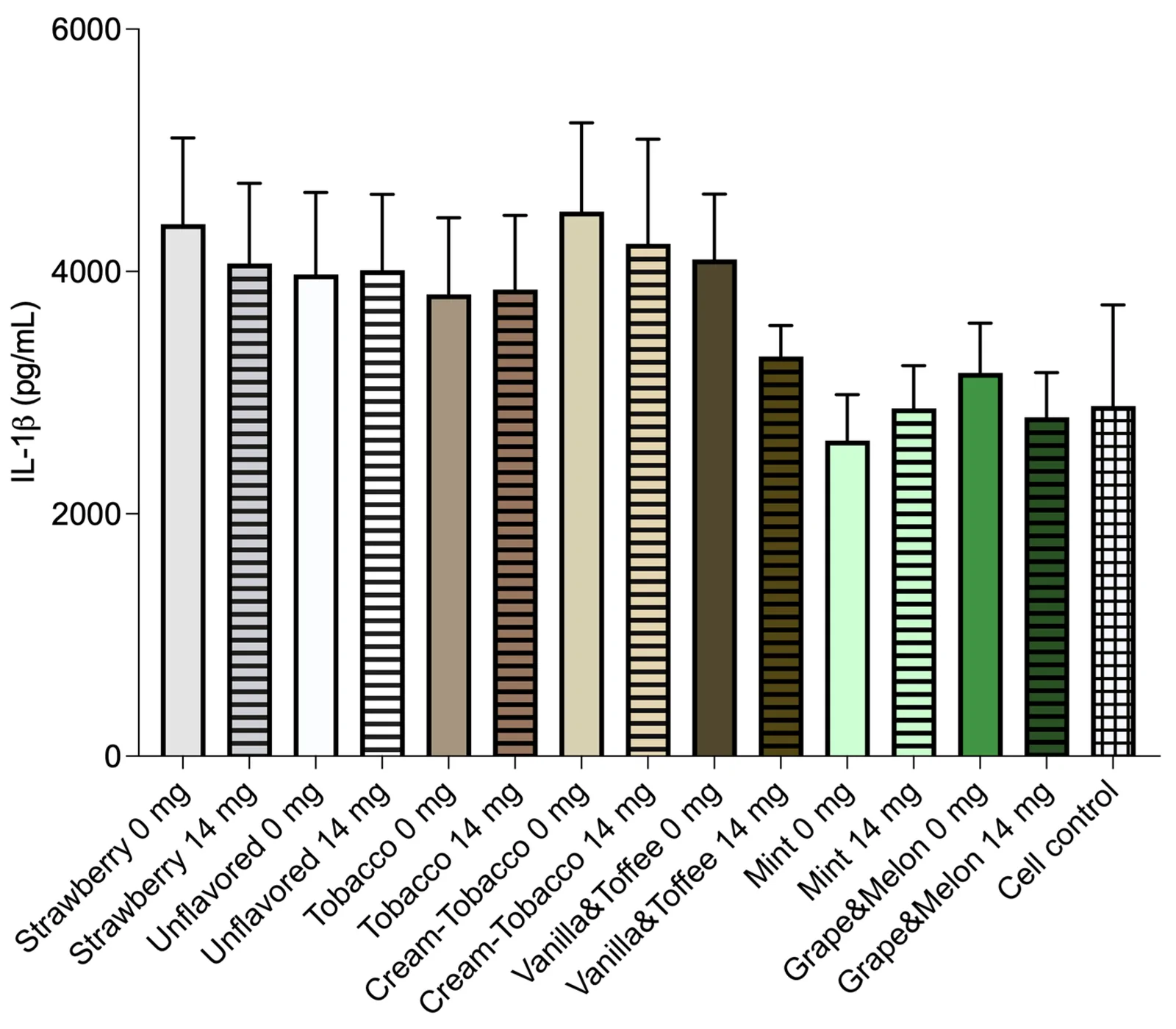
IL-1β secretion from human neutrophils following exposure to e-cigarette aerosol extracts in the presence of *Streptococcus mutans* or only *S. mutans* (cell control). Data are presented as the mean ± SEM (n = 8).

**Figure 8:**
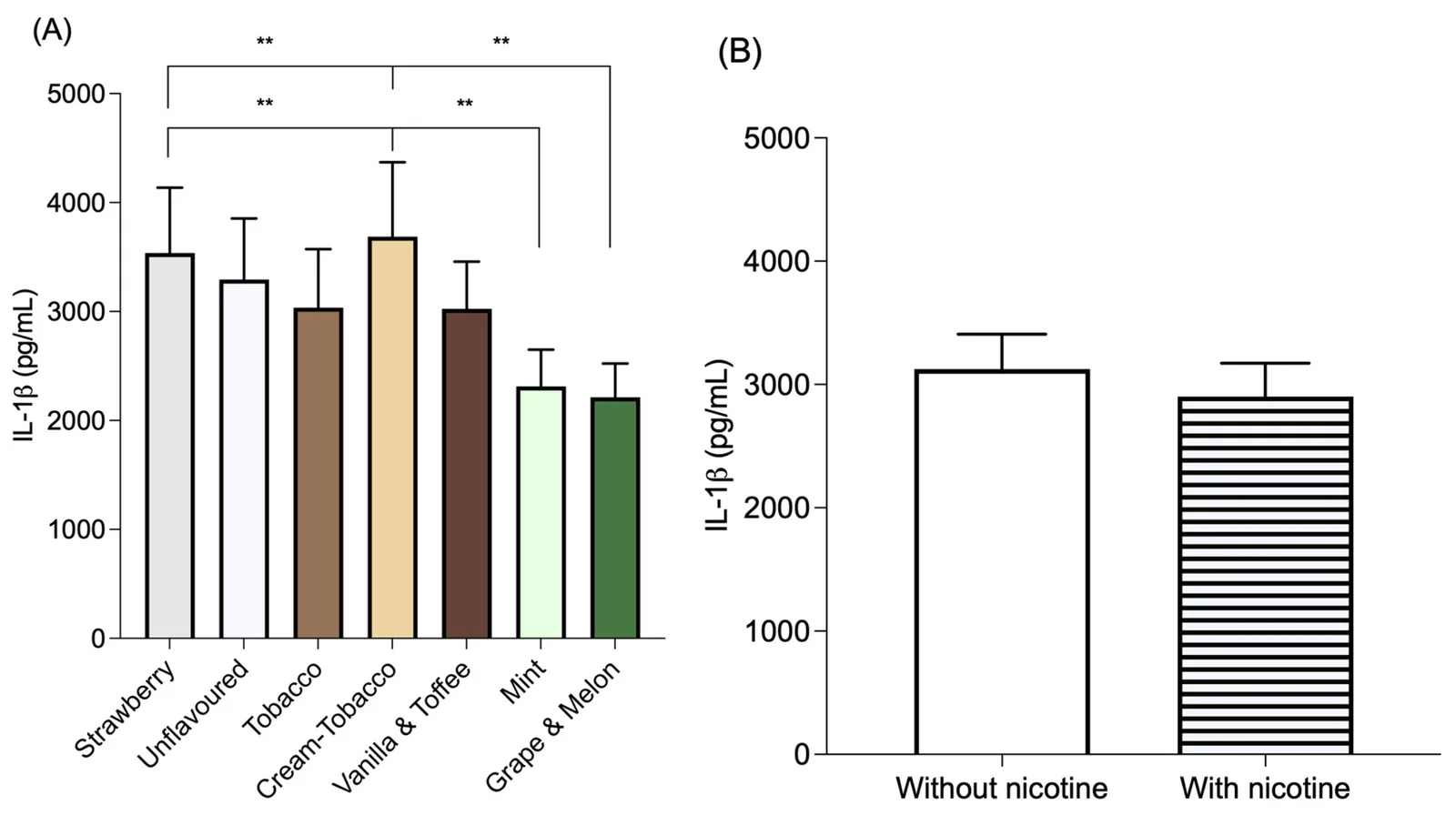
IL-1β secretion from human neutrophils following exposure to different e-cigarette aerosol extract flavors (**A**) with and without nicotine (**B**) in the presence of *Streptococcus mutans*. Data are presented as the mean ± SEM. The level of significance is indicated as ** 0.01 (n = 8).

An optimization experiment was initially performed to determine the most appropriate dilutions (1:64 and 1:512) for the subsequent experiments. Pairwise comparisons indicated that mint, grape, and melon flavors exhibited lower IL-1β levels compared with some other flavors. No group showed any statistically significant differences compared with the control group (neutrophils stimulated with *S. mutans*) (Figure 7). However, when the data were grouped based on flavor, significant differences were observed (Figure 8A).

Overall, mint, grape, and melon consistently exhibited the lowest IL-1β levels among the tested samples. When the data were grouped based on nicotine content, the nicotine-free extracts showed slightly higher IL-1β levels compared with those containing nicotine, but the difference was not significant (Figure 8B).

**Figure 9:**
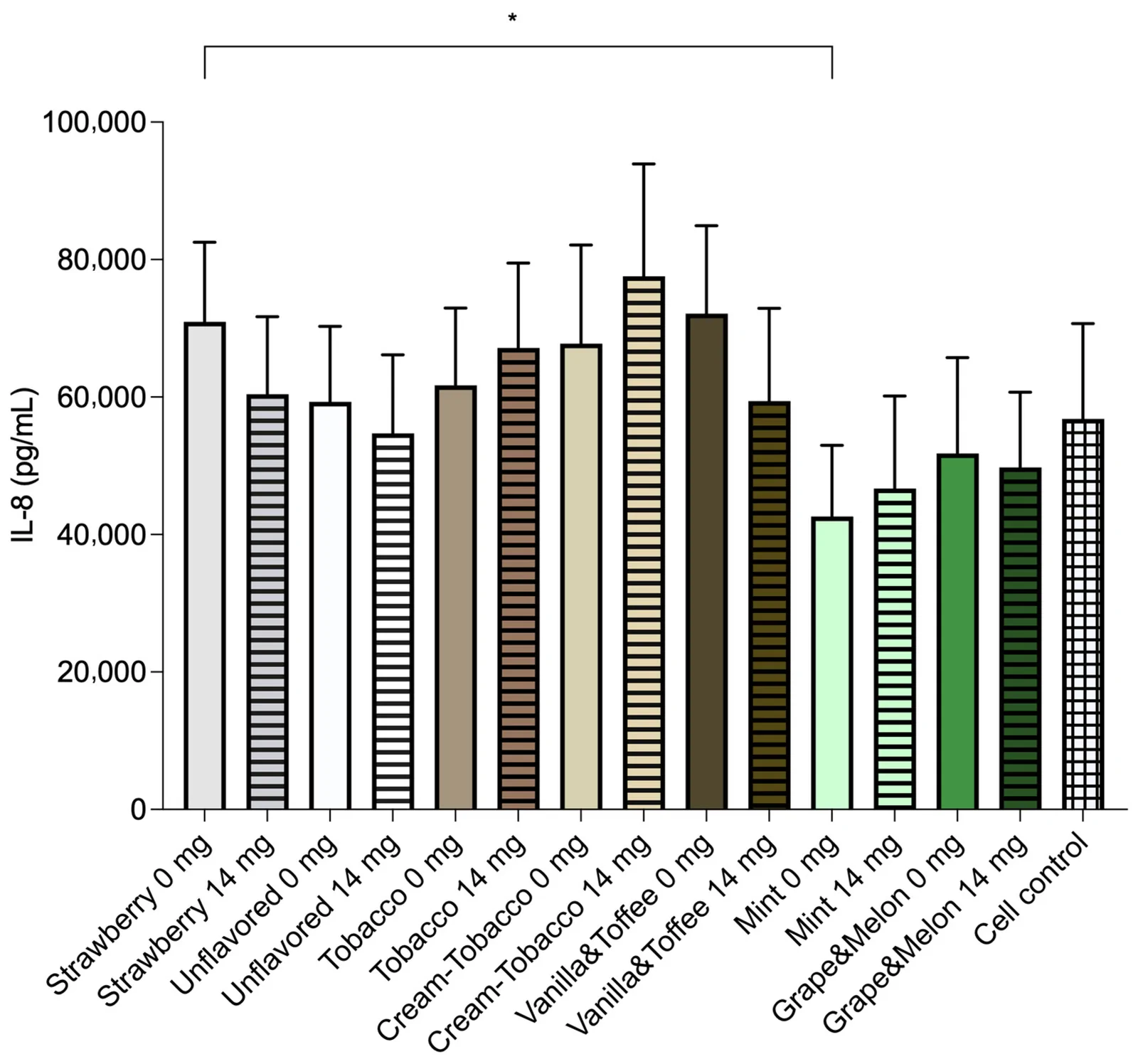
IL-8 secretion from human neutrophils following exposure to e-cigarette aerosol extracts in the presence of *Streptococcus mutans*. Data are presented as the mean ± SEM. The level of significance is indicated as * 0.05 (n = 8).

**Figure 10:**
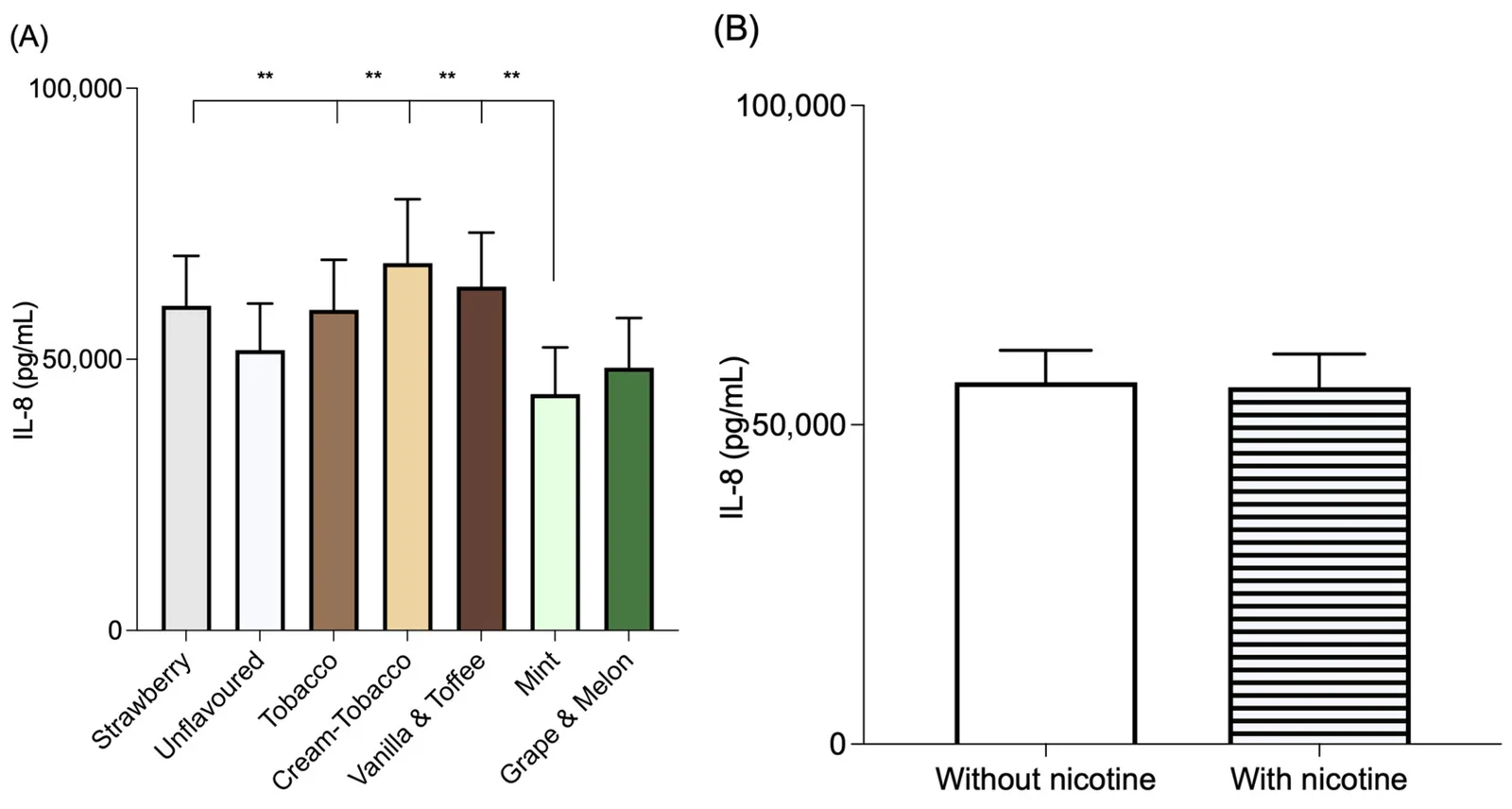
IL-8 secretion from human neutrophils following exposure to different e-cigarette aerosol extract flavors (**A**) with and without nicotine (**B**) in the presence of *Streptococcus mutans*. Data are presented as the mean ± SEM. The level of significance is indicated as ** 0.01 (n = 8).

For IL-8, the nicotine-free mint flavor induced significantly lower IL-8 secretion compared with the nicotine-free strawberry flavor (Figure 9). This observation was further supported when the data were grouped according to flavor category, where mint-flavored extracts resulted in significantly lower IL-8 release than strawberry-, cream-tobacco-, and vanilla and toffee-flavored extracts (Figure 10A). In contrast, when the data were grouped based on nicotine content, no significant differences were observed between extracts with and without nicotine, indicating that nicotine did not significantly influence IL-8 release under the experimental conditions used in this study (Figure 10B).

### 3.5. Chemical Characterization of E-Cigarette Aerosol Extracts

GC-MS analysis was performed to characterize the chemical composition of the aerosol extracts generated from the investigated e-liquids. A total of 24 compounds were identified across the analyzed samples, including nicotine, flavoring agents, aromatic compounds, esters, alcohols, and solvents. The identified compounds included menthol, levomenthol, eucalyptol, vanillin, ethyl vanillin, piperonal, trimethylpyrazine, methoxyphenol, triacetin, dibenzylamine, and triethyl citrate.

The chemical profiles differed among flavor categories. Mint-flavored products were characterized by high relative abundances of menthol, levomenthol, eucalyptol, and the cooling agent ethyl-disopropyl-acetamide. Tobacco- and creamy tobacco-flavored products showed higher relative abundances of trimethylpyrazine, benzyl alcohol, 2-acetylpyrrole, vanillin, and ethyl vanillin compared with the other flavor categories. Strawberry-flavored products exhibited a distinct ester-rich profile characterized by compounds such as hexanoic acid ethyl ester and hexenyl acetate, whereas grape and melon products were more strongly associated with cooling agents than with fruity esters.

**Figure 11:**
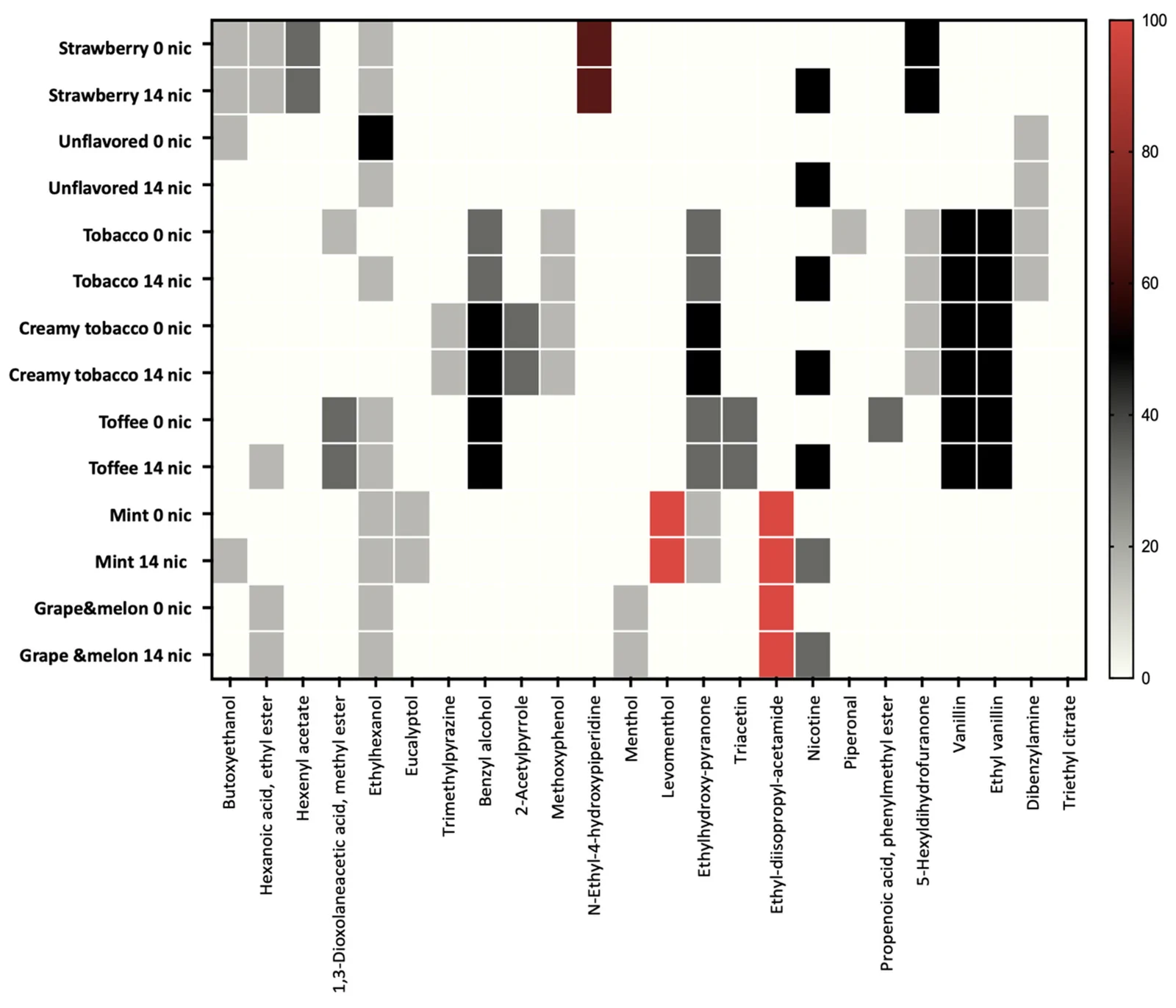
Heatmap showing the distribution of compounds identified in e-cigarette aerosol extracts by GC-MS analysis. Columns represent the identified compounds, and rows represent individual aerosol extracts. Color intensity reflects normalized semi-quantitative abundance indices derived from the GC-MS data evaluation and is intended for relative comparison between samples rather than quantitative determination of compound concentrations. The heatmap illustrates differences in chemical composition among the investigated flavor categories.

To visualize similarities and differences in chemical composition among the products, the detected compounds were summarized in a heatmap (Figure 11). Distinct patterns in compound distribution were observed according to flavor category, indicating that flavor was a major determinant of aerosol composition. In contrast, no similarly distinct pattern was apparent according to nicotine content, suggesting that the observed variation in chemical composition was more closely associated with flavor category than with nicotine content.

## 4. Discussion

This study investigated the effects of e-cigarette aerosol extracts on human neutrophil function, focusing on cell viability, oxidative burst capacity, and cytokine production in response to *S. mutans*. The main findings indicate that e-cigarette aerosol extracts did not compromise neutrophil viability under the experimental conditions used, but affected antimicrobial functions involved in host defense against oral bacteria. Exposure to certain e-liquid flavorings reduced oxidative responses in neutrophils stimulated with *S. mutans*, whereas only modest, flavor-dependent effects were observed for the pro-inflammatory cytokines IL-1β and IL-8.

Assessment of LDH release showed no increase in cytotoxicity across all tested conditions, regardless of flavor or nicotine content. These findings suggest that neutrophil viability was not affected following exposure to e-cigarette aerosol extracts. Interestingly, LDH levels in treated samples were slightly lower than those observed in the unstimulated control samples, although the biological significance of the findings remains unclear. Similar effects have been described following exposure to bacterial components such as lipopolysaccharide (LPS), which can prolong neutrophil survival [22,23]. However, whether a similar mechanism could account for the present findings cannot be determined from the LDH assay alone, and the observed reduction in LDH release should therefore be interpreted with caution.

Despite preserved viability, neutrophil functional responses appeared to be affected. The reduced intracellular ROS production observed in neutrophils exposed to *S. mutans* suggests an impairment of the oxidative burst, a key mechanism in neutrophil-mediated bacterial killing [12]. However, luminol-enhanced chemiluminescence measures ROS generation and does not directly assess bacterial phagocytosis or killing. Therefore, the reduced ROS responses observed in this study should not be interpreted as direct evidence of impaired phagocytic or bactericidal capacity. The present findings indicate that certain tobacco- and dessert-related flavors, all of which are common, reduced ROS generation. This may indicate a reduced antimicrobial response that could potentially affect bacterial clearance. As ROS generation represents a central component of neutrophil-mediated antimicrobial defense, the observed reduction suggests that certain e-cigarette aerosol extracts may interfere with normal innate immune responses to oral microorganisms such as *S. mutans*. Although bacterial clearance was not directly assessed in the present study, these findings indicate a potential modulation of host defense mechanisms involved in the control of cariogenic bacteria.

These findings are consistent with previous studies demonstrating that e-cigarette aerosols can impair innate immune functions without inducing overt cytotoxicity [24,25]. Notably, the suppressive effect on ROS production was flavor-dependent, with tobacco and dessert flavors showing the most pronounced impact, whereas fruit and mint flavors did not significantly alter oxidative responses [26,27]. This is consistent with the hypothesis that flavoring agents, in addition to nicotine, may contribute to the modulation of immune cell function. NADPH oxidase is a central component of neutrophil ROS generation [28], raising the possibility that flavor-dependent modulation of oxidative responses could involve pathways regulating NADPH oxidase activation. However, NADPH oxidase activity was not directly assessed in the present study. To further explore these flavor-dependent effects, chemical characterization of the e-cigarette aerosol extracts was performed using GC-MS analysis. Distinct differences in chemical composition were observed between flavor categories. Mint-flavored products were characterized by menthol, levomenthol, and eucalyptol, whereas tobacco- and dessert-related products showed a higher relative abundance of aromatic flavoring compounds including vanillin, ethyl vanillin, benzyl alcohol, 2-acetylpyrrole, and trimethylpyrazine. Interestingly, the strongest suppression of neutrophil ROS production was observed in the tobacco and dessert flavor groups, while mint and fruit flavors showed little effect. GC-MS profiles provide a possible chemical context for these flavor-dependent differences. Tobacco- and dessert-related products, which showed the strongest reduction in ROS production, also showed higher relative abundances of aromatic flavoring compounds such as vanillin, ethyl vanillin, benzyl alcohol, 2-acetylpyrrole, and trimethylpyrazine. These compositional differences could potentially influence pathways involved in neutrophil ROS generation, including processes related to NADPH oxidase activation. In contrast, mint-flavored products, which had a smaller effect on ROS production, were characterized by high relative abundances of menthol, levomenthol, and eucalyptol. Although the present study was not designed to establish causal relationships between individual compounds and biological responses, these findings suggest that differences in flavor composition may contribute to the observed modulation of neutrophil oxidative responses. These associations should be regarded as hypothesis-generating and warrant further investigation using targeted approaches to evaluate the biological effects of individual compounds.

The interaction between neutrophils and *S. mutans* is particularly relevant in the context of dental caries. Certain *S. mutans* strains have been shown to activate neutrophils in different ways, which may influence the efficiency of neutrophil-mediated clearance and contribute to bacterial persistence within oral biofilms [29]. Such effects may alter host–microbe interactions and contribute to conditions favorable for the development and progression of dental caries.

In contrast to the effects observed on oxidative responses, e-cigarette aerosol extracts had only modest effects on IL-1β and IL-8 release in neutrophils stimulated with *S. mutans*. Although no consistent effect of nicotine was observed, some flavor-dependent differences were detected, particularly for IL-8, where mint-flavored extracts induced lower cytokine release compared with several other flavor categories. These findings suggest that certain flavoring agents may modulate neutrophil responses to *S. mutans* and alter innate immune signaling pathways involved in host–microbe interactions, rather than inducing a pronounced pro-inflammatory response. IL-1β and IL-8 play important roles in inflammatory signaling and neutrophil recruitment and are often elevated in oral inflammatory conditions [13]. However, the relatively modest changes observed in the present study suggest that vaping aerosols may exert subtle immunomodulatory effects rather than robust inflammatory activation. Interestingly, slightly lower IL-1β levels were observed in nicotine-containing extracts. Although these differences were not statistically significant, nicotine has previously been reported to exert anti-inflammatory effects through activation of the cholinergic anti-inflammatory pathway [12]. Further studies are needed to determine whether such mechanisms contribute to the modulation of neutrophil responses following e-cigarette exposure. Overall, the findings suggest that e-cigarette aerosol exposure primarily affects antimicrobial effector functions rather than cytokine-mediated inflammatory responses. Previous pulmonary exposure models have shown that chronic exposure to e-cigarette aerosols can result in dysregulated chemokine production [27,30,31,32], indicating that exposure duration and frequency may influence immune responses to vaping aerosols.

Importantly, these findings suggest that the observed changes in ROS production and cytokine release are not associated with cell death but rather reflect functional alterations in viable immune cells. Previous studies investigating gingival epithelial cells and fibroblasts have similarly not consistently observed cytotoxic effects at low or moderate exposure levels [31,32].

Taken together, the findings indicate that e-cigarette aerosol extracts may alter neutrophil function, selectively affecting antimicrobial defense mechanisms without inducing cytotoxicity or marked inflammatory activation. This selective pattern of immune modulation may have important implications for oral health. Impaired neutrophil responses could reduce the host’s ability to control cariogenic bacteria, potentially contributing to biofilm persistence and increased susceptibility to dental caries or other oral diseases.

Several limitations should be considered. First, the in vitro design does not fully replicate the complex conditions of the oral environment, including salivary flow, biofilm structure, and chronic exposure patterns. Second, the acute exposure model may not reflect the cumulative effects of long-term vaping. Furthermore, the 25% aerosol extract concentration used in the functional experiments represents experimental in vitro exposure and cannot be directly translated into an equivalent oral exposure level in e-cigarette users. Actual exposure in the oral cavity is likely to vary according to factors such as puffing behavior, aerosol deposition, salivary dilution, and clearance. Third, although neutrophils from multiple independent donors were used, inter-individual donor variability may have influenced the magnitude of the observed responses. The paired statistical approach accounted for within-donor comparisons; however, the relatively limited number of donors may restrict the generalizability of the findings. Fourth, *S. mutans* was used as a single bacterial model relevant to dental caries. The observed neutrophil responses may therefore not be representative of responses to other oral microorganisms or polymicrobial biofilms. Finally, as previously mentioned, the oral environment is considerably more complex than the present experimental system and includes interactions among saliva, epithelial cells, immune cells, microorganisms, and biofilm communities, as well as dynamic aerosol deposition and clearance. These factors should be considered when extrapolating the present in vitro findings to e-cigarette exposure in vivo.

## 5. Conclusions

Within the limitations of this in vitro study, the results indicate that flavored e-cigarette aerosols do not induce significant neutrophil cytotoxicity but may alter oxidative burst activity in a flavor-dependent manner. The absence of cytotoxic effects does not necessarily indicate immunological safety, as functional alterations in neutrophil responses may affect host defense against oral pathogens.

Although only minor alterations in IL-1β and IL-8 production were observed, the observed flavor-dependent variations suggest subtle modulation of inflammatory signaling pathways. In combination with the reduced oxidative responses, these findings point toward functional immune modulation rather than overt inflammatory activation.

Further longitudinal and in vivo studies are needed to determine whether repeated or chronic exposure to e-cigarette aerosols could influence oral host–microbe interactions and contribute to the development or progression of oral disease.

## Figures and Tables

**Table 1:** Details of the e-cigarette liquid products used in this study.

Flavor	Conc Nic, Country	Conc Nic, Country
Cream-Tobacco	0 Nic, UK	14 mg/mL Nic, UK
Grape and Melon	0 Nic, UK	14 mg/mL Nic, UK
Mint	0 Nic, UK	14 mg/mL Nic, UK
Strawberry	0 Nic, UK	14 mg/mL Nic, UK
Tobacco	0 Nic, UK	14 mg/mL Nic, UK
Unflavored	0 Nic, SE	14 mg/mL Nic, SE
Vanilla Toffee	0 Nic, UK	14 mg/mL Nic, UK

Country: manufacturing country for the product; UK: (Preston, United Kingdom); SE: (Tibro, Sweden); Nic: nicotine.

## Data Availability

The datasets generated and/or analyzed during the current study are available from the corresponding author upon reasonable request.
